# *Mycoplasma gallisepticum *infection in the grey partridge *Perdix perdix*: outbreak description, histopathology, biochemistry and antioxidant parameters

**DOI:** 10.1186/1746-6148-7-34

**Published:** 2011-07-08

**Authors:** Frantisek Vitula, Lucie Peckova, Hana Bandouchova, Miroslav Pohanka, Ladislav Novotny, David Jira, Jiri Kral, Karel Ondracek, Jitka Osickova, Dagmar Zendulkova, Katerina Rosenbergova, Frantisek Treml, Jiri Pikula

**Affiliations:** 1 Department of Veterinary Ecology and Environmental Protection, Faculty of Veterinary Hygiene and Ecology, University of Veterinary and Pharmaceutical Sciences, Brno, Czech Republic; 2 Centre of Advanced Studies, Faculty of Military Health Sciences, University of Defence, Hradec Kralove, Czech Republic; 3 Department of Pathological Morphology and Parasitology, Faculty of Veterinary Medicine, University of Veterinary and Pharmaceutical Sciences, Brno, Czech Republic; 4 Department of Infectious Diseases and Microbiology, Faculty of Veterinary Medicine, University of Veterinary and Pharmaceutical Sciences, Brno, Czech Republic

## Abstract

**Background:**

The grey partridge is an important game bird in Europe that has declined considerably over the last decades. The production and release of farm-bred birds can be threatened by infectious agents. The objective of this study was to describe the outbreak, pathology, and blood and tissue biochemical responses in a flock of grey partridges naturally infected with *Mycoplasma gallisepticum*.

**Results:**

Morbidity and mortality rates were 100% and 60%, respectively. Necropsy revealed an accumulation of caseous exudate within the infraorbital sinuses, tracheitis, pneumonia and airsacculitis. There were significant increases in activities of lactate dehydrogenase, creatine kinase and amylase, and levels of total protein and glucose in *Mycoplasma*-infected birds when compared to control. Catalase showed significantly lower activity in the heart, lungs, liver and gonads of *Mycoplasma*-infected birds. Glutathione-S-transferase activity was elevated in the eye and the associated infraorbital sinus and kidneys, and decreased in the liver. Decreased levels of reduced glutathione were found in the heart, kidneys, liver and gonads. The activity of glutathione reductase was lower only in the lungs. Compared to healthy birds, mycoplasmosis in the grey partridge caused significant differences in the level of lipid peroxidation in lungs and plasma (p < 0.05), while the ferric reducing antioxidant power was lower in the heart and kidneys (p < 0.01). Significant correlations among responses of the antioxidant parameters were found namely in the heart, lungs, spleen, liver and plasma. There were also numerous significant inter-tissue correlations of all the studied antioxidant parameters.

**Conclusions:**

The present study demonstrates the high susceptibility of grey partridges to natural infection by *M. gallisepticum*, the severity of the disease based on histopathology, and the modulation of blood chemical profiles and oxidative stress-associated parameters in the avian hosts, thus enhancing the understanding of the pathogenesis of mycoplasmosis in birds. Moreover, the reported reference values can be useful for the evaluation of the state of health in grey partridges.

## Background

The grey partridge *Perdix perdix*, a famous bird native to Europe and introduced to many parts of the world, has shown a marked population decline throughout Europe since the second half of the 20^th ^century [1]. Factors driving this decline include a sharp decrease in chick survival due to pesticide use, habitat loss due to agricultural intensification and mechanisation, lower hatching success, and increased predation [2]. As this avian species is both of conservation and commercial game management concern, captive-bred grey partridges are widely used for repopulation purposes [3,4]. The release of farm-bred birds presents some risks that can combine and result in high mortality rate in the reintroduced population [5]. Game bird reintroductions have been reported to fail because of behavioural deficiencies, post-release predation, lower disease resistance, disease outbreaks, and genetic differences between the released and the wild birds [3,5-7]. Birds originating from infected captive flocks may introduce new agents into the wild populations or suffer from adverse effects of the parasite burdens they carry [8,9]. The release of other game birds, such as *Phasianus colchicus *pheasants and *Alectoris rufa *red-legged partridge, can also prove harmful for grey partridges due to competition and shared pathogens [2].

An outbreak of mycoplasmosis occurred at a game bird farm (Moravia, Czech Republic) producing birds for release and affected a flock of breeding grey partridges in 2010. Laboratory diagnosis confirmed *Mycoplasma gallisepticum*, the most economically significant mycoplasma pathogen of poultry [10,11]. This organism has already been reported to cause disease in game birds including pheasants, chukar and red-legged partridges, bobwhite quail, Japanese quail and peafowl [11-15]. Recent isolations of the agent from passerines with conjunctivitis and the spread of the infection throughout eastern USA and Canada have warranted its classification among emerging infectious diseases in wildlife [16,17].

*Mycoplasma *infections are known to be associated with oxidative damage of host cells and tissues due to reactive oxygen species generated by both the immune system of the host and the bacterium as its primary virulence factor [18-24]. Resistance to oxidative stress, therefore, seems to be crucial for mycoplasmas to survive within the host [21]. Responses of the host cell antioxidant defence systems to the human pathogen *Mycoplasma pneumoniae *have been studied extensively *in vitro *using human cell cultures [18-20,23,24]. *M. gallisepticum *infection of cultured chicken embryo fibroblasts has also demonstrated that attachment of viable mycoplasmas to cells is crucial to decrease catalase activity and that this lower enzyme activity may be important for the development of cytopathic effects [22].

With the exception of one study on the oxidative stress and alterations of antioxidant status in blood samples from goats infected with *Mycoplasma agalactiae *[25], there are no *in vivo *reports concerning other animal species and birds in particular. While clinical signs, lesions, pathogenicity, epizootiology, laboratory diagnosis and control of the *M. gallisepticum *infection in game birds are well described [11-15], little is known about the effect of the pathogen on the avian host regarding oxidative stress and biochemistry in association with histopathological findings. The molecular and cellular events that lead to the development of lesions and clinical disease are still obscure [10]. Thus, the objective of this study was to describe the outbreak and evaluate normal blood and tissue biochemical parameters as well as biochemical responses and pathology in a flock of grey partridges naturally infected with *M. gallisepticum*.

## Methods

### Experimental birds and samples collected

One-year-old captive-bred adult grey partridges were used in the study. The birds were kept at a game bird farm (Moravia, Czech Republic). Control healthy birds (44 males and 44 females) were blood sampled for normal biochemistry and evaluation of gender differences. A total of 5 suspected *Mycoplasma-*infected pairs of birds showing pronounced clinical signs of respiratory disease were blood sampled and then sacrificed by decapitation in order to collect samples for bacterial laboratory diagnosis and organs including the heart, eye and the associated infraorbital sinus, central nervous system, lungs, kidney, spleen, liver and gonads for histopathology and measurements of antioxidant parameters. A control group of 5 healthy pairs were also sampled in this way. The study was performed in compliance with the laws for the protection of animals against cruelty as approved by the Ethical Committee of the University of Veterinary and Pharmaceutical Sciences Brno, Czech Republic.

### Bacterial diagnosis (*Mycoplasma *culture and identification)

Clinical specimens were collected and cultured as previously recommended [26]. Swabs from the trachea, nares and conjunctival sacs, the inflammatory content of infraorbital sinuses and a piece of lung tissue were plated onto the mycoplasma agar and inoculated into the broth for the isolation of avian mycoplasmas [27], as modified by Bradbury [28]. Samples were incubated at 37°C in an atmosphere of 5% CO_2_. Broths were examined daily for colour change in the pH indicator and plates every 2-3 day for colony growth. The *Mycoplasma *spp. isolates were further identified using the nested polymerase chain reaction (PCR). The presence of *M. gallisepticum *DNA was detected using a nested PCR based on the amplification of the 16S rRNA gene sequence specific for *M. gallisepticum *(accession number M22441). Primers for the 1^st ^and 2^nd ^PCR were selected using the Vector NTI Suite 5.5 (INFORMAX) and synthesised on a commercial basis by the Generi Biotech company (Czech Republic). Sequence of primers for the 1^st ^PCR reaction are Sn: 5'ATG CTG AGA GGT AGA ATA ACC 3' and Asn: 5'CCA CCT TAC GGA TTT GC 3'; for the 2^nd ^PCR Sn: 5'GGC GAA GGC GAG GAC TTG GG 3' and Asn: 5'GCA CCG AAG TAT TCG CTC CGA CAC 3'. The NucleoSpin Tissue Kit (Machery - Nagel, Germany) was used to isolate the total DNA from the sample as described by the producer. Both PCR reactions were performed in 20 μl of the reaction mixture in a Biometra *T-*personal thermocycler (Germany). The reaction mixture contained PPP Master Mix (i.e., 200 μM of each dNTP, 2.5 mM MgCl_2_, 2U Taq Purple DNA polymerase; Top Bio, Czech Republic) and primers in the concentration of 25 pmol/μl. Amplification in both reactions included 30 cycles of denaturation at 94°C for 35 s, annealing at 49°C (the 1^st ^PCR) or 66°C (the 2^nd ^PCR) for 25 s and elongation at 72°C for 90 s. The PCR product (volume 10 μl) gained in the 2^nd ^PCR was analysed by electrophoresis in the 2% agarose gel stained with ethidium bromide and visualised using an UV transilluminator. The specific PCR product of 130 bp was compared with the molecular weight marker O'GeneRuler DNA Ladder Mix (Fermentas International Inc., USA).

Apart from the mycoplasma culture, routine bacteriological examination was performed. Samples collected from the liver, spleen, kidneys, lungs, infraorbital sinuses and air sacs were aerobically incubated at 37°C for 48 hours on blood agar and MacConkey agar. The bacterial growths were then identified by standard methods.

### Serological tests

Control healthy birds and partridges suspected from *Mycoplasma *infection were serologically examined for avian respiratory infections including 1) Newcastle disease (haemagglutination inhibition test with VLDIA039 HAG-NDL live antigen for use in the HI test and VLDIA053 HAR-NDL monospecific antiserum for use as a positive control in the HI test; GD-Animal Health Service, Deventer, the Netherlands), 2) avian influenza (AI Ab enzyme-linked immunosorbent assay ELISA; IDEXX Laboratories, Inc., Westbrook, Maine, USA), 3) infectious bronchitis (IBV Ab ELISA; IDEXX Laboratories, Inc., Westbrook, Maine, USA), 4) infectious laryngotracheitis (agar gel immunodiffusion with VLDIA014 AGA-ILT live antigen for use in the AGID test for infectious laryngotracheitis and VLDIA022 AGP-ILT monospecific infectious laryngotracheitis antiserum for use as a positive control in the AGID test and VLDIA030 SPF-CH chicken negative control serum for use as a negative control in most poultry assays; GD-Animal Health Service, Deventer, the Netherlands), 5) avian rhinotracheitis (APV Ab ELISA; IDEXX Laboratories, Inc., Westbrook, Maine, USA), 6) avian chlamydiosis (Chlamydia complement fixation test; Institute Virion Ltd., Zurich, Switzerland) as well as antibodies against 7) *M. gallisepticum *(MG Ab ELISA; IDEXX Laboratories, Inc., Westbrook, Maine, USA).

### Histopathology

Specimens of heart, eye and the associated infraorbital sinus, central nervous system, lungs, kidney, spleen, liver and gonads (ovaries and testes) were collected and placed in 10% buffered formalin during autopsy, and were treated using a routine histological technique and embedded in paraffin. Sections of 5 μm thicknesses were made from the paraffin blocks and these were stained with haematoxylin and eosin.

### Plasma biochemistry

Blood (1 ml) was collected from the right jugular vein using the Omnican^® ^40 (Braun, Germany). Whole blood was placed in heparinised tubes (Leciva inj., Prague), centrifuged immediately, and plasma was removed and frozen (-20°C). Within a few days, plasma was analysed using an automated analyser (SPOTCHEM™ EZ SP-4430, ARKRAY, Japan) for aspartate aminotransferase (μkat/l), alkaline phosphatase (μkat/l), lactate dehydrogenase (μkat/l), creatine kinase (μkat/l), alanine aminotransferase (μkat/l), total protein (g/l), total cholesterol (mmol/l), high-density lipoprotein cholesterol (mmol/l), triglycerides (mmol/l), glucose (mmol/l), amylase (μkat/l), uric acid (mmol/l), calcium (mmol/l) and phosphorus (mmol/l).

### Antioxidant parameters

The antioxidant parameters were assayed in tissues (heart, eye and the associated infraorbital sinus, central nervous system, lungs, kidney, spleen, liver, ovaries and testes) and plasma samples were collected at the time of autopsy and kept at -80°C until use. Biochemicals, enzymes and other chemicals used in the study were purchased from Sigma-Aldrich (Prague, Czech Republic) and were of the highest available commercial grade. The tissues were homogenised on ice using a mechanical homogeniser (100 mg of tissue in 1 ml) in 50 mM potassium phosphate buffer (KH_2_PO_4 _with 1 mM EDTA, pH 7.4) for assessment of catalase (CAT) activity and in phosphate buffered saline (PBS, pH 7.2) for other parameters. The postmitochondrial supernatant was collected after centrifugation (30 min at 30,000 *g *at 4°C for CAT and 15 min at 10,000 *g *at 4°C for the other parameters) and stored frozen at -80°C until biochemical analyses. The methods for the assessment of most of the biochemical markers measured are described in our previous article [29]. Briefly, glutathione-*S*-transferase (GST) activity was measured spectrophotometrically using 1-chloro-2,4-dinitrobenzene. The concentration of reduced glutathione (GSH) was determined using 5,5'-dithiobis-2-nitrobenzoic acid (DTNB) as a chromogen. Activity of glutathione reductase (GR) was determined from the rate of NADPH oxidation. The level of lipid peroxidation in avian tissues was assessed as total thiobarbituric acid reactive species (TBARS). Activity of CAT was evaluated spectrophotometrically at 240 nm in cuvettes as the rate of hydrogen peroxide breakdown in the mixture containing 0.09% hydrogen peroxide in 50 mM TRIS/0.1 mM EDTA buffer [30]. The protein concentrations were determined by the method using the Folin-Ciocalteu phenol reagent. The GENios spectrophotometric microplate reader (Tecan Group, Switzerland) was used to measure the absorbance in all assays and the VARIAN CARY 50 Bio spectrophotometer (Varian, USA) was used for measuring absorbance of solutions in cuvettes. The total antioxidant capacity was measured using the ferric reducing antioxidant power assay (FRAP). The FRAP assay was performed as described previously [31], with minor modifications. In the first round, the FRAP reagent was prepared as a mixture of 2.5 ml of 10 mM 2,4,6-tris(2-pyridyl)-*s*-triazine (TPTZ) in 40 mM HCl and 2.5 ml of 20 mM FeCl_3 _in 25 ml of 0.1 M acetate buffer pH 3.6. The freshly prepared FRAP reagent was incubated at 37°C for 10 minutes. The volume of 30 μl of the tissue or plasma sample was mixed with 200 μl of the FRAP reagent and then with distilled water up to 1 ml. After 10 min of incubation, the mixture was centrifuged at 10,000 *g*. A blank sample was prepared in the same way as described above but saline solution was used instead of the tissue or plasma sample. Absorbance of the supernatant was measured at 593 nm against the blank.

### Statistical analysis

Statistica for Windows^® ^7.0 (StatSoft, Tulsa, OK, USA) was used to compare different groups by one-way analysis of variance (ANOVA) and post-hoc analysis of means by the LSD test. The homogeneity of variances was tested by Levene's test. Non-homogenous parameters, as determined by Levene's test, were log-transformed prior to analysis. In these cases, the non-parametric Kruskal-Wallis test was used for comparing the groups. A *post-hoc *power analysis was conducted to show that the sample size of 10 healthy and 10 infected birds in this study provided sufficient statistical power for comparing tissue biochemical parameters. Values of *p *< 0.05 and *p *< 0.01 were considered statistically significant and highly significant, respectively, for all tests. Spearman rank order correlations were used to assess the relationships among the measured parameters.

## Results

### Outbreak description

**Figure 1:**
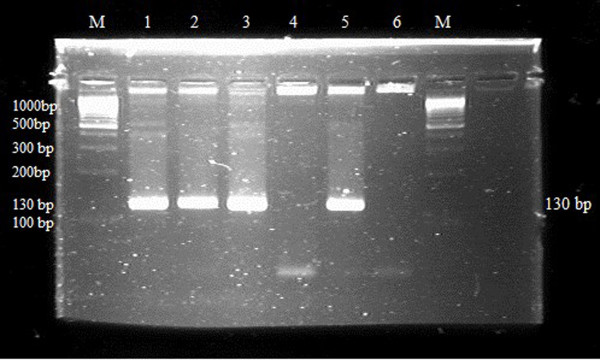
**Detection of *M. gallisepticum *by nested PCR**. M = Molecular size marker, 100 bp DNA ladder (O'GeneRuler™ DNA Ladder Mix, Fermentas International Inc., USA). 1 to 3 - positive samples, 4 - a negative sample, 5 - positive control, 6 - negative control.

**Figure 2:**
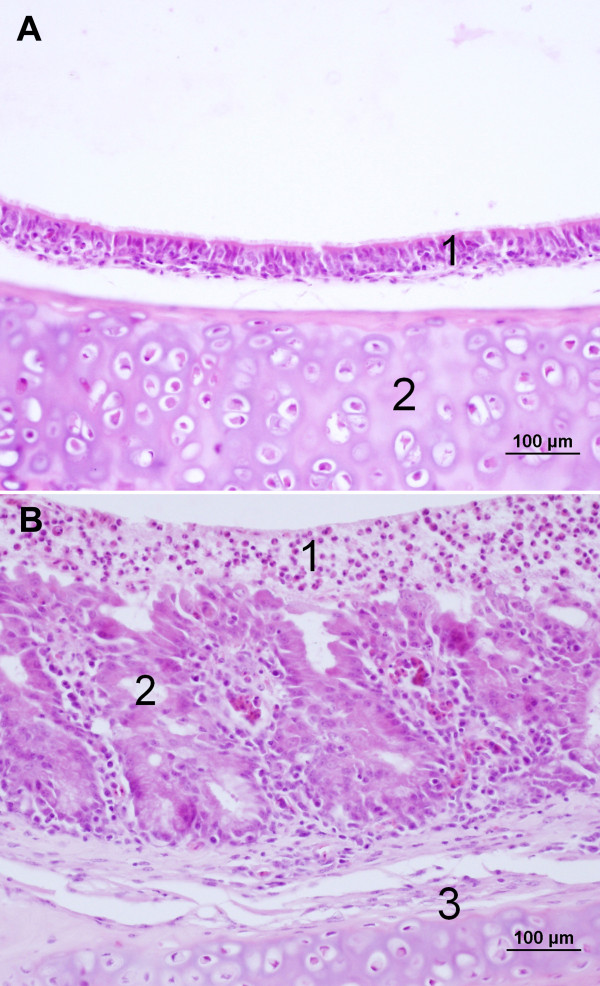
**Comparison of normal tracheal tissue and tracheitis with inflammatory cell infiltration in *M. gallisepticum*-infected birds**. Figure 2A: Trachea of a partridge from the control group. Normal mucosa lined with a pseudo-stratified columnar epithelium with kinocilia (1) and a fibrocartilaginous layer (2) are presented. No signs of inflammation were observed in any of the tracheal layers. Epithelial detachment is artificial due to sample processing. H&E stain. Figure 2B: Trachea of a partridge infected with *M. gallisepticum*. The inflammatory exudate, composed mostly of heterophils, is adhered to the epithelium (1). The epithelium is moderately hyperplastic with loss of the kinocilia and lamina propria is moderately infiltrated by lymphocytes and plasma cells (2). The fibrocartilaginous layer (3) is composed of the dense connective tissue mildly infiltrated by lymphocytes and plasma cells, and the perichondrium and hyaline cartilage. H&E stain.

**Figure 3:**
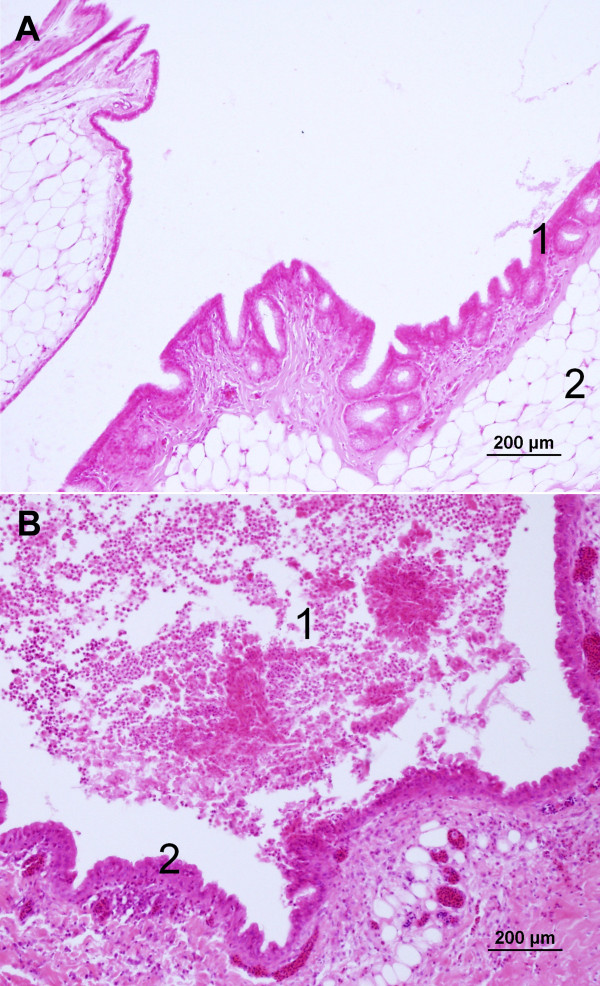
**Comparison of normal and mycoplasmal infraorbital sinus demonstrating the serious nature of pathology in *M. gallisepticum*-infected birds**. Figure 3A: The infraorbital sinus of a partridge from the control group. The sinus is lined with a simple columnar epithelium (1). The sinus is surrounded by adipose tissue (2). H&E stain. Figure 3B: The infraorbital sinus of a partridge infected with *M. gallisepticum*. The sinus is richly filled with a dense purulent exudate (1). The epithelium is mildly hyperplastic and the lamina propria is focally infiltrated by lymphocytes and plasma cells (2). H&E stain.

**Figure 4:**
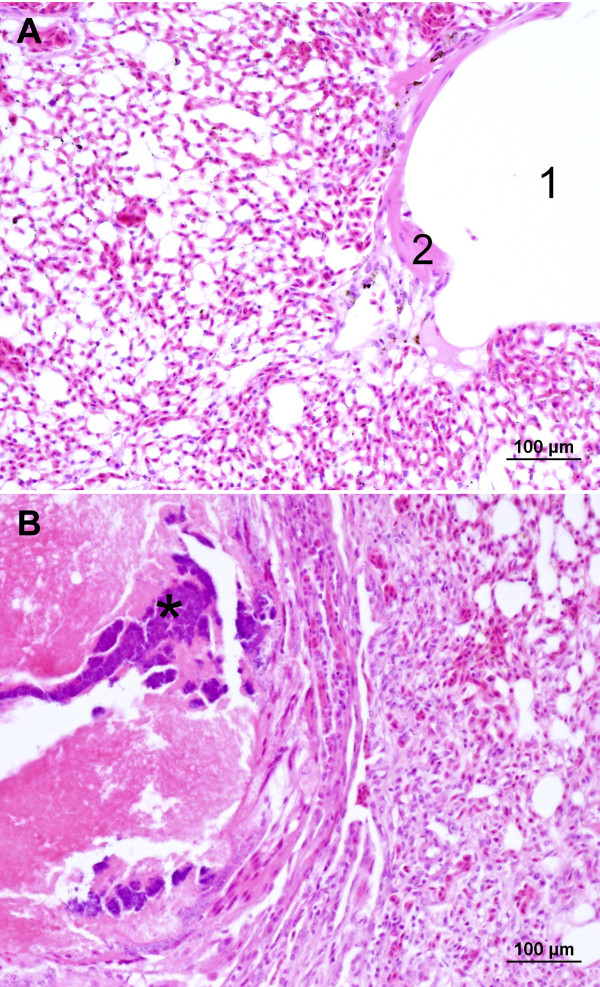
**Comparison of normal lungs and pneumonia with necrotic areas and bacterial colonies in *M. gallisepticum*-infected birds**. Figure 4A: The lung parenchyma of a partridge from the control group. Parabronchial lumen (1) and wall with bundles of smooth muscle tissue (2) is surrounded by air and blood capillaries. H&E stain. Figure 4B: A focus of necrotic pneumonia with secondary bacterial colonisation (*) surrounded by a thin layer of heterophils and lymphocytes in a partridge infected with *M. gallisepticum. *H&E stain.

An outbreak of respiratory disease occurred in a flock of adult grey partridges kept at a game bird farm (Moravia, Czech Republic). The infection developed gradually in April to May 2010, culminated by morbidity of 100% and 60% (i.e., a total of 68 partridges) died within three to four weeks of appearance of clinical signs. Clinical signs included nasal and ocular discharge and dyspnoea with laboured open-mouth breathing, eyelid and infraorbital sinus swelling, lethargy, poor intake of feed and weight loss. There was a marked drop in egg production. Culture revealed *Mycoplasma *spp. in tracheal swabs only. A slight colour change (acidity) appeared in the broth medium on the 4^th ^day and first centered mycoplasma colonies were recognized on plates on the 5^th ^day of incubation. The isolated strain was further identified as *M. gallisepticum *using the nested PCR methods (Figure 1). Bacteriological culture of the liver, spleen, kidneys, lungs, small intestinal content, infraorbital sinuses and air sacs yielded *Escherichia coli *from infraorbital sinuses, lungs and air sacs. Serological tests for Newcastle disease, avian influenza, infectious bronchitis, infectious laryngotracheitis, avian rhinotracheitis and avian chlamydiosis were negative in birds included in the control healthy and *Mycoplasma-*infected groups of partridges. The titre of antibodies against *M. gallisepticum *ranged from 1404 to 5884 in the *Mycoplasma-*infected group. Necropsy revealed an accumulation of caseous exudate within the infraorbital sinuses, tracheitis, pneumonia and airsacculitis. Histopathological findings can be summarised as severe purulent infraorbital sinusitis, moderate purulent tracheitis with marked epithelial hyperplasia, moderate to severe purulent bronchitis and extensive purulent and necrotic pneumonia with secondary bacterial colonisation of necrotic areas. Figures 2A and 2B show the comparison of the normal tracheal tissue and tracheitis with inflammatory cell infiltration in *M. gallisepticum*-infected birds. Likewise, Figures 3A and 3B of normal and mycoplasmal infraorbital sinus, respectively, demonstrate the serious nature of pathology in this tissue and Figures 4A and 4B show the comparison of normal and necrotic lung tissues.

### Biochemistry and antioxidant parameters

Table 1 presents normal plasma chemical profiles in healthy adult grey partridges as well as values measured in *Mycoplasma*-infected birds. Comparison of healthy males and females revealed gender differences in creatine kinase, total protein and high-density lipoprotein cholesterol. Significant increases in the biochemical parameters of *Mycoplasma*-infected birds, when compared to controls, included activities of lactate dehydrogenase, creatine kinase and amylase, and levels of total protein and glucose.

**Table 1:** Differences in biochemical parameters between healthy control and *Mycoplasma*-infected partridges

Parameters	Groups of birds	
	**Healthy control birds**	***Mycoplasma*-infected birds**

**AST (μkat/l)**	4.88 ± 1.93	4.84 ± 1.35

**ALP (μkat/l)**	3.79 ± 1.64	3.42 ± 1.75

**LDH (μkat/l)**	2.55 ± 1.35	8.20 ± 3.47**

**CK (μkat/l)**	11.58 ± 13.30 (M 17.21 ± 15.60; F 5.94 ± 7.05**)	28.88 ± 18.13**

**ALT (μkat/l)**	0.69 ± 0.13	0.62 ± 0.26

**TP (g/l)**	38.62 ± 7.99 (M 36.52 ± 5.36; F 40.76 ± 9.59*)	54.16 ± 9.64**

**T-CHOL (mmol/l)**	3.17 ± 1.00	3.70 ± 0.83

**HDL-CHOL (mmol/l)**	2.30 ± 0.75 (M 2.54 ± 0.80; F 2.06 ± 0.63**)	1.95 ± 0.40

**TG (mmol/l)**	1.31 ± 0.65	1.02 ± 0.80

**GLU (mmol/l)**	18.90 ± 2.60	21.68 ± 4.91*

**AMY (μkat/l)**	3.50 ± 1.47	5.98 ± 1.52**

**UAC (μmol/l)**	608.46 ± 418.91	918.66 ± 445.45

**Ca (mmol/l)**	2.63 ± 0.25	2.84 ± 0.22

**P (mmol/l)**	0.98 ± 0.41	1.09 ± 0.50

Values represent mean ± SD; n = 88 in healthy control birds (44 males and 44 females), n = 10 in *Mycoplasma*-infected birds; * = p < 0.05, ** = p < 0.01; significant gender differences shown where appropriate. AST = aspartate aminotransferase, ALP = alkaline phosphatase, LDH = lactate dehydrogenase, CK = creatine kinase, ALT = alanine aminotransferase, TP = total protein, T-CHOL = total cholesterol, HDL-CHOL = high-density lipoprotein cholesterol, TG = triglycerides, GLU = glucose, AMY = amylase, UAC = uric acid, Ca = calcium, P = phosphorus, M = male, and F = female.

Differences in antioxidant parameters between healthy control and *Mycoplasma*-infected grey partridges are shown in Table 2. While catalase showed significantly lower activity in the heart, lungs, liver and gonads of *Mycoplasma*-infected birds, glutathione-S-transferase was elevated in the eye and the associated infraorbital sinus and kidney, and decreased in the liver. Decreased levels of reduced glutathione were found in the heart, kidney, liver and gonads of birds affected by mycoplasmosis and the activity of glutathione reductase was lower only in the lungs. Compared to healthy birds, mycoplasmosis in the grey partridge caused significant differences in the level of lipid peroxidation measured as the total thiobarbituric acid reactive species in avian lungs and plasma. The ferric reducing antioxidant power was lower in the heart and kidneys.

**Table 2:** Differences in antioxidant parameters between healthy control and *Mycoplasma*-infected partridges

Parameters	Tissues	Groups of birds	
		**Healthy control birds**	***Mycoplasma*-infected birds**

**CAT**	Eye + infraorbital sinus	61.80 ± 23.37	48.91 ± 16.63

	Heart	66.90 ± 25.95	39.28 ± 4.40*

	Central nervous system	41.57 ± 9.09	44.18 ± 20.07

	Lungs	61.33 ± 16.16	38.34 ± 11.34*

	Kidney	48.18 ± 14.89	50.75 ± 21.54

	Spleen	57.19 ± 20.00	40.19 ± 8.81

	Liver	63.15 ± 14.38	43.36 ± 6.43*

	Gonads (ovaries/testes)	62.62 ± 22.30	36.95 ± 11.37*

	Plasma	59.67 ± 12.88	47.72 ± 24.24

**GST**	Eye + infraorbital sinus	19.49 ± 12.55	46.06 ± 19.41*

	Heart	134.16 ± 43.02	215.66 ± 98.39

	Central nervous system	53.21 ± 34.50	80.41 ± 32.21

	Lungs	67.70 ± 35.61	69.44 ± 34.74

	Kidney	236.40 ± 72.85	362.33 ± 64.05*

	Spleen	25.08 ± 18.82	71.66 ± 67.29

	Liver	389.80 ± 92.82	254.33 ± 98.20*

	Gonads (ovaries/testes)	134.16 ± 43.02	215.68 ± 98.37

	Plasma	645.83 ± 90.52	461.80 ± 157.78

**GSH**	Eye + infraorbital sinus	2.00 ± 0.04	1.929 ± 0.15

	Heart	1.78 ± 0.04	1.33 ± 0.03**

	Central nervous system	2.03 ± 0.17	1.91 ± 0.10

	Lungs	1.76 ± 0.05	1.73 ± 0.17

	Kidney	1.82 ± 0.20	1.44 ± 0.17**

	Spleen	1.79 ± 0.06	1.83 ± 0.05

	Liver	2.35 ± 0.30	1.59 ± 0.12**

	Gonads (ovaries/testes)	1.91 ± 0.17	1.49 ± 0.32*

	Plasma	212.52 ± 4.04	232.93 ± 17.27*

**GR**	Eye + infraorbital sinus	2.46 ± 0.87	3.48 ± 1.51

	Heart	28.45 ± 25.82	7.27 ± 2.34

	Central nervous system	7.82 ± 3.49	5.49 ± 2.73

	Lungs	57.55 ± 13.07	26.34 ± 9.31**

	Kidney	36.44 ± 8.84	39.03 ± 9.00

	Spleen	28.99 ± 12.22	24.16 ± 16.51

	Liver	53.32 ± 15.68	36.70 ± 9.55

	Gonads (ovaries/testes)	10.93 ± 6.74	8.57 ± 3.58

	Plasma	529.20 ± 392.81	321.54 ± 84.73

**TBARS**	Eye + infraorbital sinus	0.14 ± 0.03	0.15 ± 0.03

	Heart	0.14 ± 0.01	0.17 ± 0.04

	Central nervous system	0.19 ± 0.04	0.18 ± 0.03

	Lungs	0.09 ± 0.02	0.14 ± 0.02*

	Kidney	0.20 ± 0.04	0.18 ± 0.04

	Spleen	0.14 ± 0.01	0.17 ± 0.04

	Liver	0.14 ± 0.01	0.17 ± 0.04

	Gonads (ovaries/testes)	0.14 ± 0.01	0.17 ± 0.04

	Plasma	216.46 ± 47.06	133.32 ± 59.80*

**FRAP**	Eye + infraorbital sinus	4.52 ± 1.31	2.76 ± 1.27

	Heart	1.96 ± 0.27	1.52 ± 0.12**

	Central nervous system	2.09 ± 0.31	2.00 ± 0.11

	Lungs	1.72 ± 0.44	1.77 ± 0.43

	Kidney	6.84 ± 0.93	3.77 ± 1.62**

	Spleen	4.52 ± 1.31	2.76 ± 1.27

	Liver	1.72 ± 0.44	1.77 ± 0.43

	Gonads (ovaries/testes)	2.47 ± 0.63	3.11 ± 1.01

	Plasma (mmol/g)	1.72 ± 0.44	1.77 ± 0.43

Values represent mean ± SD; n = 10 in healthy control birds (5 males and 5 females), n = 10 in *Mycoplasma*-infected birds (5 males and 5 females); * = p < 0.05, ** = p < 0.01. CAT = catalase (μmol H_2_O_2_/min/mg protein), GST = glutathione-*S*-transferase (μkat/g), GSH = reduced glutathione (μmol/g), GR = glutathione reductase (μkat/g), TBARS = total thiobarbituric acid reactive species (μmol/g), FRAP = ferric reducing antioxidant power assay (μmol/g).

As shown in Table 3, significant correlations among responses of the antioxidant parameters were found namely in the heart, lungs, spleen, liver and plasma. Reduced glutathione positively correlated with ferric reducing antioxidant power, glutathione reductase and catalase in the heart and with glutathione reductase, glutathione-S-transferase and catalase in the liver. There was also a positive correlation between glutathione reductase and catalase and ferric reducing antioxidant power in lungs and spleen, respectively, while glutathione-S-transferase and ferric reducing antioxidant power were in a close positive relationship with catalase both in the liver and plasma. Table 3 also presents numerous significant inter-tissue correlations of all the studied antioxidant parameters. Apart from glutathione-S-transferase in lungs and plasma and reduced glutathione in heart and plasma, all other inter-tissue correlations were positive.

**Table 3:** Spearman rank order correlations of antioxidant parameters in tissues and plasma

Tissue	Correlated parameters	+/- correlation	P-level
**Heart**	GSH&FRAP	+	< 0.01

	GSH&GR	+	< 0.05

	GSH&CAT	+	< 0.05

**Lungs**	GR&CAT	+	< 0.05

**Spleen**	GR&FRAP	+	< 0.01

**Liver**	GSH&GR	+	< 0.05

	GSH&GST	+	< 0.01

	GSH&CAT	+	< 0.01

	GST&CAT	+	< 0.05

**Plasma**	FRAP&CAT	+	< 0.05

**Parameters**	**Correlated tissues**	**+/- correlation**	**P-level**

**CAT**	Heart & gonads	+	< 0.01

	Heart & liver	+	< 0.05

	Eye & lungs	+	< 0.05

	CNS & kidney	+	< 0.05

	Lungs & liver	+	< 0.05

	Lungs & plasma	+	< 0.05

	Kidney & plasma	+	< 0.05

	Gonads & liver	+	< 0.01

**GST**	Heart & eye	+	< 0.05

	Heart & gonads	+	< 0.001

	Heart & spleen	+	< 0.05

	Eye & gonads	+	< 0.01

	Eye & spleen	+	< 0.01

	Lungs & plasma	-	< 0.05

	Gonads & spleen	+	< 0.05

**GSH**	Heart & kidney	+	< 0.01

	Heart & gonads	+	< 0.05

	Heart & liver	+	< 0.001

	Heart & plasma	-	< 0.01

	Eye & gonads	+	< 0.05

	Kidney & liver	+	< 0.01

	Gonads & liver	+	< 0.05

**GR**	Heart & plasma	+	< 0.05

**TBARS**	Heart & lungs	+	< 0.05

	Heart & gonads	+	< 0.001

	Heart & spleen	+	< 0.001

	Heart & liver	+	< 0.001

	Lungs & gonads	+	< 0.05

	Lungs & spleen	+	< 0.05

	Lungs & liver	+	< 0.05

**FRAP**	Eye & spleen	+	< 0.001

	Lungs & plasma	+	< 0.001

	Liver & plasma	+	< 0.001

CAT = catalase, GST = glutathione-*S*-transferase, GSH = reduced glutathione, GR = glutathione reductase, TBARS = total thiobarbituric acid reactive species, FRAP = ferric reducing antioxidant power assay, eye = eye + the associated infraorbital sinus, CNS = central nervous system, gonads = ovaries or testes, n = 20.

## Discussion

The outbreak of respiratory disease occurred at the peak of the laying period four months after the introduction of new partridges, i.e., the supposed source of infection in the game bird farm. The long incubation period is in agreement with reports on birds remaining asymptomatic until they are stressed [11]. The disease broke out long after removing the birds from the one-month quarantine and the stress of the laying period was probably the factor triggering clinical manifestation. Pheasants and partridges are known to harbour many fast-growing mycoplasmas, making the isolation of the slower-growing *M. gallisepticum *difficult [13]. In the present study, however, the isolation and identification of this mycoplasma species was straightforward. The high titres against *M. gallisepticum *together with its cultural and PCR identification witness for an ongoing systemic *M. gallisepticum *infection. While the high morbidity in the grey partridge flock corresponds with rates observed both in poultry and other avian species infected by *M. gallisepticum *as the sole pathogen, the high mortality is rather typical for mycoplasmosis complicated by some other infectious agent [11-15,32]. Indeed, *E. coli *was cultured from infraorbital sinuses, lungs and air sacs of some mycoplasmal birds and there were bacterial colonies in the lungs upon investigation by histopathology. It has been experimentally shown that combined stressors exert enhanced effects in birds [33], and, apart from *E. coli*, other bacteria and viruses such as *Pasteurella multocida *and infectious bronchitis virus, respectively, may be implicated in synergistic respiratory infections with *M. gallisepticum *[13-15]. Serology, however, excluded other common respiratory infections in birds included in the control healthy and *Mycoplasma-*infected groups of partridges.

Mycoplasmosis affecting birds in the laying period results in reduced egg production and quality [11]. The quantity of eggs laid by the grey partridge breeding flock decreased abruptly following the disease outbreak and eggs produced were not incubated to prevent contamination of the hatching device. It was, therefore, not possible to evaluate the biological quality of the clutch using such characteristics as viability and hatchability. Since an immune challenge decreases the reproductive allocation to the egg in the grey partridge [34], mycoplasmas are egg transmitted [11], and mycoplasmosis can also induce salpingitis in birds [35], the total effect of hatching (i.e., the percentage of chicks hatching from all eggs set) would certainly be very poor if the eggs were incubated.

Gross and microscopic pathology was specifically used to demonstrate the severity of the disease in grey partridges affected by *M. gallisepticum *in this biochemical study. Previous papers have reported sinusitis and bilateral swelling of the infraorbital sinuses as the most outstanding feature, airsacculitis in 46%, and tracheitis and lung lesions in 36 and 21% of cases, respectively [11,14,15]. Comparing the effects of experimental intranasal infection with *M. gallisepticum *and *Mycoplasma imitans *in red-legged partridges, nasal and sinus exudates were found in both groups, while tracheal exudates and airsacculitis were only seen in the *M. gallisepticum *infection [14]. As the culture revealed *M. gallisepticum *in tracheal swabs and changes in the grey partridge were very similar to those mentioned above, pathological findings of the present study were in agreement with those observed by other authors [11,14,15].

As shown in Table 1, gender differences in healthy control birds do not interfere with the interpretation of significant responses in plasma chemical profiles of *Mycoplasma*-infected partridges. The results indicate that diagnosis of avian mycoplasmosis solely based on clinical biochemical parameters is not possible. They can, nevertheless, be used for the evaluation of the general health status in mycoplasmal birds [36]. One would expect lower total protein and glucose due to starvation and weight loss in the *Mycoplasma*-infected group. Contrary to this, there was an increase in total plasma protein and glucose levels, probably as a consequence of inflammation or dehydration in the former parameter and stress in the latter. A response somewhat different from that in partridges was seen in caprine mycoplasmal pneumonia because total protein level was found to be lower, while the glucose level was increased [37]. Amylase catalyses the hydrolysis of polysaccharides, it is associated with glycaemia and its increase corresponds with the observed higher levels of glucose [38]. Both enzymes activities of which were significantly increased in mycoplasmal partridges, i.e., lactate dehydrogenase and creatine kinase, are closely associated. Lactate dehydrogenase is found in skeletal and cardiac muscle, liver, kidney, bone and erythrocytes and elevations can be observed with disruption of any of these. Distinguishing the source of lactate dehydrogenase elevation is based on measuring creatine kinase that originates mainly in skeletal and cardiac muscle. Elevated lactate dehydrogenase levels (three-fold) with concurrent elevation in creatine kinase (two-fold) in the present study are thus suggestive of skeletal or cardiac muscle damage [36].

The research presented here showed modulations of antioxidant parameters, the total antioxidant capacity and oxidative damage in the form of lipid peroxidation associated with the respiratory disease caused by a natural infection with *M. gallisepticum *of grey partridges. Oxidative stress is an unspecific biochemical process involved in the adverse action of many stressors. There is a clear association between oxidative stress and immune responses of birds to infectious agents [39]. Endogenous antioxidant defences of an enzymatic and non-enzymatic nature are essential for the control of reactive-molecular-species-mediated oxidative damage of biomolecules [40]. Interestingly, changes in oxidative stress parameters were not restricted to the respiratory apparatus of mycoplasmal birds, but also occurred in non-respiratory organs and plasma. Similarly, extrapulmonary complications were recognised in humans with *M. pneumoniae *infection [41]. Statistical analysis also revealed significant correlations among responses of the oxidative stress parameters in the heart, lungs, spleen, liver and plasma, and numerous inter-tissue correlations of all the studied oxidative stress parameters. Correlations among the oxidative stress parameters illustrate the complex character of the response and interdependence of parameters. Significant correlations among the studied parameters observed in the liver confirm the major metabolic role of this organ in birds. It would be an interesting issue for future studies to evaluate the relationship between the tissue mycoplasmal burden and oxidative stress parameters, as has been done in other bacterial infections [42].

Hydrogen peroxide and superoxide radicals produced by mycoplasmas are coupled with endogenous toxic oxygen molecules generated by the host cells to induce oxidative stress that then results in host cell damage [24,41]. It has been suggested that the pathogenesis of mycoplasmosis comprises the following sequence of events: (a) adherence of mycoplasmas to host cells; (b) generation of superoxide and hydrogen peroxide by the microorganisms and their introduction into host cells; (c) irreversible inhibition of host cell catalase by intracellular reactive-oxygen-species accumulation; and (d) oxidative damage to vital cell constituents [18,19,22]. In agreement with the above mentioned *in vitro *findings, catalase showed significantly lower activity in the heart, lungs, liver and gonads of *Mycoplasma*-infected partridges.

Significant changes were found for the glutathione-related parameters. Glutathione-S-transferase was elevated in the eye and the associated infraorbital sinus and kidney, and decreased in the liver. Decreased levels of reduced glutathione were in the heart, kidney, liver and gonads and the activity of glutathione reductase was lower only in the lungs of birds affected by mycoplasmosis. These results correspond basically with data on the protective role of the glutathione redox cycle and its adaptive responses observed in cultured fibroblasts and mice infected with *M. pneumoniae*, respectively [20,23]. Similarly, decreases in plasma glutathione concentrations and glutathione peroxidase activity were reported in goats naturally infected with *M. agalactiae *[25].

It is possible to evaluate the total antioxidant capacity of biological fluids using the ferric reducing antioxidant power assay as a clinical marker of oxidative stress. Non-enzymatic antioxidants such as ascorbic acid, uric acid, bilirubin, vitamin E, α-tocopherol and albumin contribute to the ferric reducing antioxidant power, the reaction is linearly related to their molar concentrations, and uric acid is estimated to make around 60% of the contribution to the plasma value [31]. Importantly, the primary route of excretion of nitrogenous waste in birds is via the formation of uric acid in the liver and its elimination by renal tubular secretion [36]. As shown in Table 1, uric acid levels were increased in the blood of mycoplasmal birds, but not significantly owing to the greater variability of data. Despite it, the total antioxidant capacity of plasma was nearly the same in both groups of birds and agreed with normal plasma values already published for the grey partridge [43]. The ferric reducing antioxidant power values were significantly lower only in the heart and kidneys of *Mycoplasma*-infected birds.

While ingested carotenoids are both ornamental pigments and antioxidants in birds, *M. gallisepticum *infection can disrupt their utilisation and result in a trade-off between immune system activation, oxidative stress and the health or sexual quality traits [44,45]. These and other diet-derived antioxidants can therefore be supplemented to feeds for *Mycoplasma*-infected captive birds as a means of supportive therapy.

Compared to healthy birds, mycoplasmosis in the grey partridge caused significant differences in the level of lipid peroxidation, i.e., a parameter of damage to membrane lipids, measured as the total thiobarbituric acid reactive species in avian lungs and plasma. Contrary to the situation in goats naturally infected with *M. agalactiae *[25], lipid peroxidation was decreased in plasma samples collected from mycoplasmal partridges. This is, however, understandable in light of the birds from the group infected by *M. gallisepticum *being able to maintain normal blood antioxidant capacity and induction of higher levels of the non-enzymatic antioxidant glutathione. As expected, avian mycoplasmosis was associated with increased lipid peroxidation in the lungs.

Mycoplasmas are considered to be extracellular pathogens. It has, however, been demonstrated recently that *M. gallisepticum *has the capability of entering nonphagocytic host cells, where it resists host defences and antibiotic therapy. This is also a mechanism of establishing chronic infections, while passage through the respiratory mucosal barrier is responsible for the ability to cause systemic infections [46]. As eradication is very difficult or even impossible once *M. gallisepticum *has been introduced, the breeding flock of grey partridges should be depopulated rather than used for repopulation [11].

## Conclusions

The present study demonstrated the high susceptibility of grey partridges to a natural infection with *M. gallisepticum*, the severity of the disease based on histopathology and the biochemical responses to mycoplasmosis in this avian host. While the reported data make a contribution to the understanding of the pathogenic mechanisms of mycoplasmal respiratory disease in birds, the normal biochemistry and antioxidant parameters of tissues and plasma may also prove useful as future references in experimental studies, and clinical or laboratory tests using grey partridges.

## Authors' contributions

FV carried out the whole study and drafted the manuscript. LP, HB, DJ, JK, KO and JO planned the study design, sampled the birds and performed biochemical evaluations. MP analysed plasma and tissue samples for antioxidant parameters and lipid peroxidation. LN evaluated histopathological findings. DZ performed mycoplasmal culture. KR examined the isolated mycoplasmas using the nested PCR. FT evaluated the outbreak of avian mycoplasmosis in the breeding flock of partridges. JP supervised the whole study, performed data analyses and drafted the manuscript. All authors read and approved the final manuscript.
